# The status of newborn screening in Africa: Situation analysis, future plans and call to action

**DOI:** 10.4102/ajlm.v14i1.2973

**Published:** 2025-09-30

**Authors:** Tumelo Satekge, Adekunle Okesina, John Anetor, Rajiv Erasmus

**Affiliations:** 1 Department of Chemical Pathology, Faculty of Health Sciences, University of Limpopo, Polokwane, South Africa; 2 Department of Chemical Pathology and Immunology, Faculty of Health Sciences, University of Ilorin, Illorin, Nigeria; 3 Department of Chemical Pathology, Faculty of Health Sciences, University of Ibadan, Ibadan, Nigeria; 4 Department of Chemical Pathology, Faculty of Health Sciences, Stellenbosch University, Cape Town, South Africa

Newborn screening (NBS) is a pivotal public health service with the aim of early detection of potential health issues in neonates shortly after birth.^1^ Specifically, biochemical NBS involves the systematic screening of newborns for a range of inherited or congenital disorders that are mostly asymptomatic at birth. Timely diagnosis and immediate treatment of these genetic disorders are essential to avert irreversible health complications resulting in lifelong morbidity and/or mortality in the paediatric population.^2^ The World Health Organization (WHO) has recommended that a policy of universal NBS be adopted in all countries and communities which have established rehabilitation services.^3^ Newborn screening has a direct linkage to the United Nations Sustainable Development Goal 3 which seeks to eliminate preventable deaths of newborns and children under 5 years of age by 2030.^4^

Newborn screening was first conceived in 1961 when Dr Robert Guthrie (the ‘Father’ of NBS) introduced the newborn bloodspot screening for phenylketonuria with a microbiological inhibition assay now known as the Guthrie test.^5^ By late 1963, 400 000 infants were screened for phenylketonuria in 29 states in the United States, detecting 39 cases at an estimated incidence 1:10 000. The success story of the NBS for phenylketonuria has led to the legislation of universal NBS being passed in 1963 in Massachusetts, followed by many other developed countries, including the United Kingdom. The legislative endorsement paved the way for the development of more assays to expand NBS and include more congenital disorders including inborn errors of metabolism (IEMs).^6^ Despite the positive impact of NBS to achieve desirable outcomes in the screened infants, low- and middle-income-countries, particularly in Africa, have not accomplished the implementation of universal NBS.

Newborn screening took a backseat for a long time in Africa as a result of prioritisation of communicable diseases, particularly HIV/AIDS and tuberculosis. The Prevention of Mother to Child Transmission and immunisation programmes have successfully reduced the infectious disease burden on the continent. Recent reports have revealed that there is now an increasing contribution of non-communicable diseases, especially congenital disorders, to infant morbidity and mortality.^2^ The existing infrastructure and resources of the impactful HIV/AIDS and immunisation programmes should provide the platform to launch and integrate NBS initiatives in African countries. A good example is dried blood spot sampling for HIV screening performed during the immunisation schedules, which can be expanded to screen for other congenital disorders.^7^

The challenges of implementing a sustainable NBS programme are well documented, and an important one of these is lack of strong political will to enable government’s endorsement and prioritisation. In most of these countries, the limited financial support from governments has led to the over-reliance on external funding sourced from non-government and humanitarian organisations. This threatens the long-term sustainability of NBS programmes, because funding from these external sources is unlikely to be a permanent guarantee.^8,9^ The Consortium of Newborn Screening in Africa initiative, in partnership with the American Society of Hematology and other organisations, have established sickle cell disease (SCD)-targeted NBS programmes in sub-Saharan African countries.^10^ The extremely high incidence of SCD in sub-Saharan Africa and its devastating clinical outcomes, as evidenced by high morbidity and mortality in the region, has largely informed the intervention of the Consortium of Newborn Screening in Africa and other non-governmental organisations, and has also attracted the attention of the WHO. The WHO Regional Office for Africa has highlighted the need for developing national policy on NBS of SCD directed at primary prevention, early detection, advocacy, surveillance and raising public awareness through community education.^11^ At the last workshop held on 04 June 2025 in Abuja, jointly organised by Revvity and the Newborn Screening Consortium Nigeria, an NBS entity based in Nigeria appealed to the Consortium of Newborn Screening in Africa to add congenital hypothyroidism to SCD screening. The workshop was a side event to the 5th Global Congress on SCD, which was held in Abuja on 03 June 2025–06 June 2025.

The success of the NBS is dependent on the programmes being fully incorporated into the routine health system processes, which may be hard to achieve. The NBS initiatives need to be structured to enable maximal follow-up of the neonates and families for confirmatory testing and initiation of treatment. Digital applications were introduced in Ghana to conveniently manage the SCD NBS data on the healthcare workers’ mobile phones, which also facilitates follow-ups. It is worth noting that Ghana and Morocco have been successful in sustaining one of the longest running NBS programmes in Africa.^9,12^ This success has been attributed to the considerable commitment and support from the local government in the building of local capacity. A notable challenge in the public health facilities is the early hospital discharges (a few hours after birth) as a result of limited maternity resources. The established reference cut-off values of biomarkers and metabolites to screen endocrinopathies and IEMs, respectively, are validated for NBS after 24 h of age. The expanded NBS in the setting of Africa will rely on research studies to derive appropriate reference cut-off values at 6 h after birth, to enable NBS prior to discharges.

The literature emerging from several African nations emphasises the point that IEMs are not exceedingly rare as often perceived. This underscores the magnitude of the problem and the need for Africa to rise to the challenge.^2,13^ Early treatment of some IEMs is often simple and extremely effective and is often by modifying the infant’s diet; for example, early withdrawal of galactose in galactosaemia, as well as the well-known low phenylalanine diet for phenylketonuria patients, can be lifesaving. However, because of a lack of awareness and paucity of sophisticated equipment such as the tandem mass spectrometry in most African states, NBS for IEMs is close to non-existent in Africa, unlike in the developed nations. An article by Anetor, Orimadegun, and Anetor^14^ is insightful in raising awareness of affordable and readily available techniques to pragmatically screen for IEMs in resource-limited settings. Additionally, the dried blood spot should be encouraged, as it can be stored and revisited as the NBS programme expands. Likewise, point-of-care testing in the forms of paper chromatography and haemoglobin isoform-specific antibodies in a lateral flow platform are cost-effective and user-friendly alternative methods of screening of SCD in remote areas.^15^ As demonstrated by NBS pilots in South Africa and other African countries, NBS of congenital hypothyroidism mandates prioritisation in Africa to promote neurodevelopment and avert long-term intellectual disability cost effectively through NBS.^16^

Community engagement with endorsement from traditional leaders has been demonstrated to yield success in some NBS initiatives by sensitively addressing the cultural beliefs that are often accompanied by stigmatisation resulting from misconceptions.^9,10^ The International Federation of Clinical Chemistry and Laboratory Medicine Task Force on Global NBS was tasked to develop NBS initiatives in low- and middle-income countries and to address the challenges that hinder the implementation of systematic NBS. One of the notable accomplishments of the Task Force is the successful development of a well-organised national NBS programme in the Philippines, which serves as a model to many low- and middle-income countries and from which Africa can gain insights.^17^ In the same vein, the African Federation of Clinical Chemistry NBS Committee was formed to raise awareness of NBS on the continent, and to encourage as many African countries as possible to develop a policy on NBS that is implementable and sustainable. In the spirit of commemorating the International NBS Day, the Committee organised a first-of-its-kind webinar on the dire need for NBS, representing all regions of Africa. With panellists carefully selected to represent the whole of Africa, the panel took the opportunity to call on all African countries to embrace universal NBS and to comply with the World Health Assembly resolutions 77 of 2024, and 78 of 2025, calling on all low- and middle-income countries to embrace and implement universal NBS.

In conclusion, it is evident that there is an urgent need for African countries to heed the call to action, drawing inspirations from developed nations that are already screening for up to 60 conditions. We can also draw lessons from other developing countries, such as the Philippines, to gain the encouragement required to implement a sustainable NBS programme. The implementation of universal NBS in Africa will require partnerships and collaborative efforts from key stakeholders to overcome the challenges and for the governments to legislate the NBS policy to combat infant morbidity and mortality on the African continent.

## References

[1] Anderson R, Rothwell E, Botkin JR. Newborn screening: Ethical, legal, and social implications. Annu Rev Nurs Res. 2011;29(1):113–132. 10.1891/0739-6686.29.113 22891501 PMC7768912

[2] Malherbe HL, Bonham J, Carrihill M, et al. Newborn screening in South Africa: The past, present, and plans for the future. Rare Dis Orphan Drugs J. 2024;3:7. 10.20517/rdodj.2023.49

[3] World Health Organization. Newborn and infant hearing screening: Current issues and guiding principles for action [homepage on the Internet]. 2010 [cited 2023 Sep 15]. Available from: https://www.who.int/publications/i/item/9789241599496

[4] Conroy N, Gilmore B. Child mortality and the sustainable development goals: A challenge and an opportunity. Ir J Med Sci. 2017;186:357–358. 10.1007/s11845-016-1463-1 27209186

[5] Therrell BL, Padilla CD, Borrajo GJ, et al. Current status of newborn bloodspot screening worldwide 2024: A comprehensive review of recent activities (2020–2023). Int J Neonatal Screen. 2024;10(2):38. 10.3390/ijns10020038 38920845 PMC11203842

[6] Levy HL. Robert Guthrie and the trials and tribulations of newborn screening. Int J Neonatal Screen. 2021;7(1):5. 10.3390/ijns7010005 33478143 PMC7838808

[7] Hsu L, Nnodu OE, Brown BJ, et al. White paper: Pathways to progress in newborn screening for sickle cell disease in sub-Saharan Africa. J Trop Dis Public Health. 2018;6(2):260. 10.4172/2329-891X.1000260 30505949 PMC6261323

[8] Wesonga RM, Awe OI. An assessment of traditional and genomic screening in newborns and their applicability for Africa. Inform Med Unlocked. 2022;32:101050. 10.1016/j.imu.2022.101050

[9] Archer NM, Inusa B, Makani J, et al. Enablers and barriers to newborn screening for sickle cell disease in Africa: Results from a qualitative study involving programmes in six countries. BMJ Open. 2022;12(3):e057623. 10.1136/bmjopen-2021-057623 PMC8915265 35264367

[10] Green NS, Zapfel A, Nnodu OE, et al. The consortium on newborn screening in Africa for sickle cell disease: Study rationale and methodology. Blood Adv. 2022;6(24):6187–6197. 10.1182/bloodadvances.2022007698 36264096 PMC9791313

[11] World Health Organization Regional Office for Africa. Sickle-cell disease: A strategy for the WHO African Region [homepage on the Internet]. 2011 [cited 2024 May 20]. Available from: https://apps.who.int/iris/handle/10665/1682

[12] Ohene-Frempong K, Oduro J, Tetteh H, Nkrumah F. Screening newborns for sickle cell disease in Ghana. Pediatrics. 2008;121(suppl_2):S120–S121. 10.1542/peds.2007-2022UUU

[13] Saadallah AA, Rashed MS. Newborn screening: Experiences in the Middle East and North Africa. J Inherit Metab Dis. 2007;30(4):482–489. 10.1007/s10545-007-0660-5 17701444

[14] Anetor JI, Orimadegun BE, Anetor GO. A pragmatic approach to the diagnosis of inborn errors of metabolism in developing countries. Afr J Lab Med. 2023;12(1): 1946. 10.4102/ajlm.v12i1.1946 37293316 PMC10244816

[15] Twum S, Fosu K, Felder RA, Sarpong KA. Bridging the gaps in newborn screening programmes: Challenges and opportunities to detect haemoglobinopathies in Africa. Afr J Lab Med. 2023;12(1): 2225. 10.4102/ajlm.v12i1.2225 38116518 PMC10729498

[16] Arrigoni M, Zwaveling-Soonawala N, LaFranchi SH, van Trotsenburg AP, Mooij CF. Newborn screening for congenital hypothyroidism: Worldwide coverage 50 years after its start. Eur Thyroid J. 2025;14(1):e240327. 10.1530/ETJ-24-0327 39812367 PMC11816049

[17] Padilla CD, Therrell Jr BL, Alcausin MMLB, et al. Successful implementation of expanded newborn screening in the Philippines using tandem mass spectrometry. Int J Neonatal Screen. 2022;8(1):8. 10.3390/ijns8010008 35225931 PMC8883932

